# Optimal pharmacological therapy for minor acute ischemic stroke: a network meta-analysis

**DOI:** 10.3389/fneur.2026.1834958

**Published:** 2026-06-24

**Authors:** Shuo Feng, Shibing Liu, Hongxia Zhao, Boru Jin, Minjie Mei

**Affiliations:** Department of Neurology, Shenyang Sujiatun District Central Hospital, Shenyang City, Liaoning, China

**Keywords:** acute ischemic stroke, antiplatelet therapy, antiplatelet therapy dual, minor, randomized controlled trial

## Abstract

**Background:**

Minor acute ischemic stroke (mAIS), typically defined as a National Institutes of Health Stroke Scale (NIHSS) score ≤ 5, accounts for over 50% of all ischemic strokes. However, the optimal pharmacological therapy for patients with mAIS remains controversial. Hence, this systematic review and network meta-analysis (NMA) was conducted to compare the effectiveness and safety of available pharmacological therapies for mAIS patients.

**Methods:**

PubMed, Embase, the Cochrane Library, and Web of Science databases were systematically searched for publications up to June 1, 2025. Randomized controlled trials (RCTs) and cohort studies investigating various antithrombotic drugs for mAIS were retrieved. The included outcome measures were functional outcomes (modified Rankin Scale [mRS] 0–1, mRS 0–2), early neurological improvement within 24 h, intracranial hemorrhage, newly diagnosed ischemic stroke within 90 days, and all-cause mortality (ACM). Bayesian NMA was performed using R 4.5.1 and STATA 15.1.

**Results:**

Eleven eligible studies, involving 26,176 patients, were included, which evaluated 9 different antithrombotic interventions. According to the NMA, single antiplatelet therapy (SAPT) (surface under the cumulative ranking curve [SUCRA] = 67.3%) and aspirin (SUCRA = 71.9%) were the most likely best interventions for achieving an mRS score of 0–1 and 0–2, respectively. Alteplase (SUCRA = 81.9%) had the highest probability of being the most effective in improving early neurological function within 24 h. Aspirin (SUCRA = 87.6%) was associated with the lowest incidence of symptomatic intracranial hemorrhage. Dual antiplatelet therapy (DAPT) represented the most likely best intervention in reducing newly diagnosed ischemic stroke within 90 days and ACM, with a SUCRA of 87.7 and 68.3%, respectively.

**Conclusion:**

Both DAPT and aspirin may represent safe and effective interventions for treating mAIS. However, individual patient circumstances should be considered in clinical decision-making. This analysis provides insights that may support the selection of individualized treatment strategies. The findings of this NMA, based on limited studies, remain to be further validated in future well-designed large-scale RCTs.

**Systematic review registration:**

https://www.crd.york.ac.uk/PROSPERO/search, identifier CRD420251238111.

## 1 Background

Acute ischemic stroke (AIS) is one of the major cerebrovascular diseases contributing to disability and mortality worldwide. The disease burden of AIS is continuously rising with the accelerated aging of the population. As reported by the World Health Organization, the global annual number of new stroke cases is over 15 million, with AIS accounting for more than 80% (1). The proportion of patients with minor acute ischemic stroke (mAIS, defined as a National Institutes of Health Stroke Scale [NIHSS] score ≤ 5) among all AIS patients is rising gradually from 43.1 to 51.7% (2). Although mAIS patients initially exhibit relatively mild neurological deficits, clinical follow-up studies indicate that approximately 10 to 30% of patients develop disability or experience neurological deterioration within 90 days of onset, suggesting that the risk of a poor long-term prognosis should not be overlooked (3). Therefore, exploring the optimal pharmacological treatment strategy for mAIS is clinically important for controlling short-term symptoms, improving long-term outcomes, and reducing the risk of recurrence.

Currently, intravenous thrombolysis (IVT) remains the core reperfusion approach for AIS patients within the time window from onset. However, for individuals with minor, non-disabling stroke, particularly those who are likely to have occluded large vessels but show mild symptoms, the benefit-to-risk ratio of such invasive treatments remains debatable. Antiplatelet therapy (e.g., aspirin, clopidogrel monotherapy, or dual antiplatelet therapy [DAPT]) is an important treatment regimen for mAIS. Anticoagulants (e.g., low-molecular-weight heparin) provide a treatment option for patients diagnosed with embolic AIS. Given the unique characteristics of mAIS patients, existing guidelines give divergent recommendations. For example, the European Stroke Organization (ESO) recommends IVT within the time window for eligible patients with minor stroke (4). While the absolute risk of symptomatic intracranial hemorrhage (sICH) with IVT is low (typically < 2%), some clinicians are concerned that the relative risk (compared to no thrombolysis) remains significant.

A living systematic review and meta-analysis conducted by Qin et al. specifically targeting patients with mAIS has shown that antiplatelet therapy is non-inferior to IVT in terms of favorable functional outcomes at 90 days, with a significantly lower risk of sICH (5). Moreover, recent clinical trials have highlighted the efficacy of DAPT (clopidogrel and aspirin) in minimizing the recurrence of stroke within 90 days after a minor stroke or high-risk transient ischemic attack (TIA) (6, 7). A Bayesian network meta-analysis (NMA) conducted by Lim et al., involving 28,148 patients with mild stroke or high-risk TIA, has shown that ticagrelor combined with aspirin has a 94% probability of being superior to other DAPT regimens in reducing the risk of recurrence in patients with mild stroke (8). This evidence has been incorporated into the 2026 American Heart Association (AHA) and American Stroke Association (ASA) Guideline for the Early Management of Patients with Acute Ischemic Stroke, which does not recommend the routine use of thrombolytic therapy for patients with non-disabling mild stroke. For patients with mild non-cardioembolic ischemic stroke (IS) (NIHSS score ≤ 5) who have not received intravenous alteplase within 24 h of onset, the guideline establishes a Class I, Level A recommendation to initiate DAPT (aspirin combined with clopidogrel) and continue treatment for 21 days (9). Furthermore, the latest evidence has indicated that patients with minor non-disabling IS treated with DAPT during the acute phase (within 4.5 h after onset) demonstrate non-inferior 90-day outcomes to patients treated with alteplase administered intravenously (10). In recent years, novel antiplatelet agents (e.g., ticagrelor) and anticoagulant therapies have been explored, further challenging the selection of treatment options. Although multiple randomized controlled trials (RCTs) and a traditional meta-analysis (11) have examined the efficacy of different antithrombotic regimens, most studies have enrolled patients with moderate-to-major stroke. Evidence specifically targeting the population with mAIS remains relatively scarce. Furthermore, traditional pairwise meta-analyses only directly compare two interventions. For example, Jiang et al.’s study only included pairwise comparisons between DAPT and tenecteplase (12). A comprehensive comparative analysis of pharmacological therapies for mAIS is currently lacking. Therefore, this systematic review and NMA sought to synthesize all relevant evidence from clinical trials to systematically compare the effectiveness and safety of various pharmacological regimens, including but not limited to single antiplatelet therapy (SAPT), DAPT, alteplase, and other anticoagulation strategies, in patients with mAIS. Our study intended to provide a tiered evidence chain that may support the selection of the optimal pharmacological regimen for mAIS patients. By ranking different therapeutic regimens in terms of efficacy and safety, this study was expected to offer evidence-based support for developing clinical guidelines and individualized treatment strategies.

## 2 Methods

We followed the Preferred Reporting Items for Systematic Reviews and Meta-Analyses (PRISMA) guidelines and their requirements for NMA (13) (Supplementary material 1) when reporting this NMA. The study protocol was registered in the International Prospective Register of Systematic Reviews (PROSPERO) (CRD420251238111).

### 2.1 Search strategy

PubMed, Embase, the Cochrane Library, and Web of Science databases were searched. The search covered publications up to June 1, 2025, and was limited to publications written in the English language. The search strategy was developed by combining subject headings and free-text words. The medical subject headings included ischemic stroke, ischemic strokes, cryptogenic ischemic stroke, cryptogenic embolism stroke, minor, mild. The search strategy is detailed in Supplementary material 2. The reference lists of published systematic reviews were additionally searched to ensure comprehensive literature coverage.

### 2.2 Inclusion and exclusion criteria

Studies were eligible for inclusion if they met all of the following criteria: (1) Study population: individuals with mAIS (defined as NIHSS score ≤ 5); (2) Interventions: intravenous thrombolytic agents (alteplase, tenecteplase), antiplatelet therapy (e.g., aspirin, clopidogrel monotherapy or DAPT), either alone or in combination; (3) Study design: RCTs and cohort studies; (4) Outcomes: primary outcomes (modified Rankin Scale [mRS] 0–1, mRS 0–2 at 90 days), secondary outcomes (early neurological improvement at 24 h [a decrease of ≥ 4 points on the NIHSS score within 72 h], intracranial hemorrhage (ICH), newly diagnosed ischemic stroke within 90 days, all-cause mortality [ACM]).

The following studies were excluded: (1) Animal or cell studies, case reports, research protocols, reviews, letters, editorials, conference proceedings, etc.; (2) Studies from which sufficient data could not be extracted for the quantitative synthesis due to incomplete reporting or unresolvable data errors; (3) Duplicate publications; (4) Studies with full text unavailable.

### 2.3 Literature screening and data extraction

Retrieved articles were imported into EndNote. Based on the above eligibility criteria, two researchers independently screened the titles and abstracts, and then the full texts. Disagreements were addressed by discussion or reassessment after consultation with a third researcher. Two researchers independently extracted the following data from the finally included studies using Excel 2016: first author, publication year, country, randomization and blinding design, interventions and controls, treatment duration, basic characteristics of participants, outcome measures, sex, age, sample size, pre-treatment NIHSS score, and definition of mAIS.

### 2.4 Quality assessment

The Cochrane Risk of Bias tool version 2.0 (RoB 2.0) (14) was employed to estimate the risk of bias in the included studies arising from 5 domains: randomization, deviation from intended interventions, missing outcome data, outcome measurement, and selective reporting of results. Two researchers independently assessed the quality of each study, assigning “low risk,” “high risk,” or “some concerns” to the 5 domains. Disagreements were addressed by discussion or reassessment after consultation with a third researcher. Results were visualized in a risk of bias plot. The quality of cohort studies was assessed by two researchers based on the Newcastle-Ottawa Scale (NOS) (15), covering patient selection, comparability of the study groups, and assessment of outcomes. Each study was assigned an NOS score of 0 to 9 stars, and studies with ≥ 6 stars were considered high-quality studies.

### 2.5 Statistical analysis

The outcomes were described as risk ratio (RR) with 95% credible interval (CrI). Given the heterogeneity among studies, the Bayesian hierarchical random-effects model was fitted for multiple comparisons of various interventions for mAIS first (16, 17). All the calculations and graphs were generated in R 4.5.1 and Stata 18.0. Markov chain Monte Carlo (MCMC) simulation was applied based on the likelihood function and some prior assumptions using Bayesian inference in R 4.5.1. A total of 500,000 iterations, including 20,000 annealing iterations, were run to ensure chain convergence (Gelman-Rubin < 1.1) (18–20). A hierarchical approach was employed for model comparison. The consistency model assumes consistency between direct and indirect evidence, while the inconsistency model allows for discrepancies between different sources of evidence through node-splitting. The deviance information criterion (DIC) was used for model selection: a difference in DIC values (ΔDIC) ≥ 5 indicated substantial inconsistency, and a node-splitting analysis was performed to identify inconsistent nodes; conversely, if ΔDIC < 5, the consistency model was selected in accordance with the Bayesian Occam’s razor principle (21). Heterogeneity was assessed using the *I*^2^ statistic, with values ≤ 25% indicating low heterogeneity, 25–50% indicating moderate heterogeneity, and >75% indicating high heterogeneity. To explore sources of heterogeneity and evaluate whether outcomes varied by study design, subgroup analyses were conducted based on study design (RCT or cohort study), NIHSS threshold (≤ 3 or ≤ 5), and quality rating (low, medium, or high). The relationships among the different interventions were depicted as network diagrams. Besides, comparison-adjusted funnel plots were drawn to assess potential publication bias (22, 23). Furthermore, surface under the cumulative ranking curve (SUCRA) values ranging from 0 to 100% were utilized to rank the investigated interventions (24, 25). Centered on relative efficacy, the SUCRA score presented the relative ranking of various interventions for a specific outcome, facilitating the fast identification of the most promising regimen. While a higher SUCRA score indicates a greater probability that an intervention ranks higher, this does not necessarily reflect real-world clinical benefits. Therefore, when interpreting its clinical significance, it is important to take absolute efficacy data into account. A comprehensive evaluation of the magnitude of differences, precision of effects, and acceptability is essential to avoid the misinterpretation of an intervention as superior merely based on a high ranking, despite that it may offer only marginal clinical benefits. League tables were created to display the pairwise comparisons of interventions within each outcome. To ensure the robustness of the study results, the convergence of the NMA was comprehensively assessed by examining the stability of trace plots and density plots. Node-splitting analysis was employed to evaluate both global and local inconsistency. A *p*-value < 0.05 was considered indicative of significant inconsistency between direct and indirect evidence, in which case an inconsistency model was employed to address the differences; conversely, a *p*-value ≥ 0.05 suggested acceptable consistency, in which case a consistency model was employed for the final analysis. Publication bias for outcomes was assessed using funnel plots and Egger’s test, with a *p*-value < 0.1 considered indicative of potential publication bias. Given the limited number of studies in most networks, this assessment should be interpreted with caution (26).

## 3 Results

### 3.1 Literature screening results

**Figure 1:**
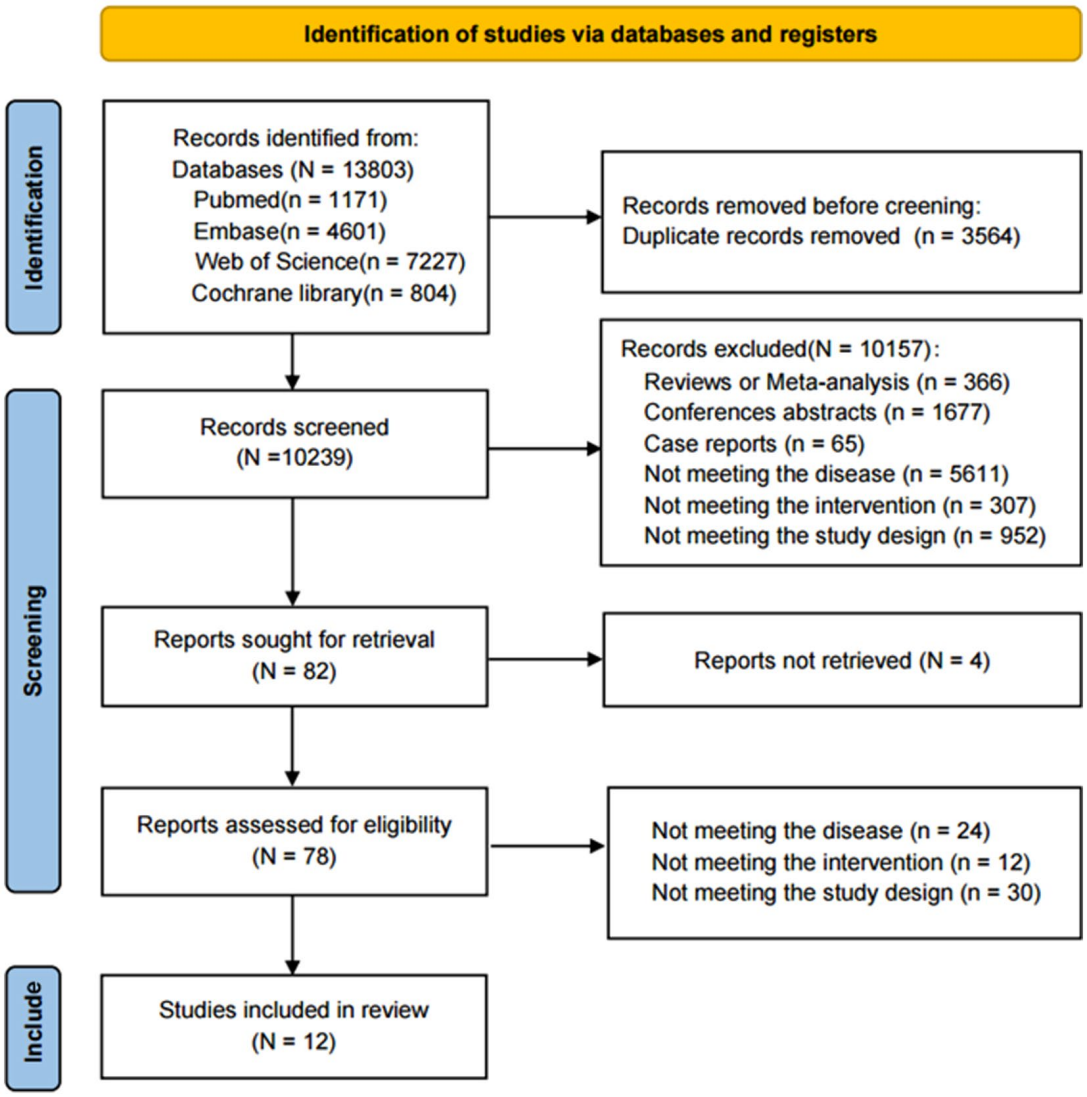
Literature screening flowchart.

A total of 13,803 articles were retrieved. Among these, 3,564 duplicates were excluded. Following an initial assessment of titles and abstracts, 10,157 articles were excluded. The remaining articles were retrieved in full-text format and screened in strict accordance with the inclusion and exclusion criteria. Finally, 12 articles were included. The screening process is depicted in Figure 1.

### 3.2 Basic characteristics of included studies

The 12 included studies (10, 27–37) originated from 7 countries (Argentina, China, South Korea, the UK, USA, Austria, and the Netherlands), involving a total of 26,176 patients. Among them, 16,373 were male, and 10,522 were female. The age of the patients ranged from 60.5 to 70.8 years. Basic characteristics of the included studies are summarized in Table 1.

**Table 1:** Baseline characteristics.

Author	Year	Country	Study design	Group	*N*	M/F	Age	Definition of mAIS	Outcome	NOS/RoB2.0
Bath PM et al.	2010	UK	RCT	DAPT	672	425/247	67.2 ± 9.2	NIHSS≤5	a.b.f	Some concerns
				Clopidogrel	688	459/229	66.7 ± 8.8			
Khatri P et al.	2018	USA	RCT	Alteplase	156	77/79	64 ± 14	NIHSS≤5	a.b.d.e.f	Low
				Aspirin	157	92/65	61 ± 13			
Lan L et al.	2020	China	Cohort study	DAPT	109	76/33	62.6 ± 10.7	NIHSS≤5	a.b.d.e	Good
				Alteplase	109	74/35	66.4 ± 11.1			
Kim S et al.	2022	Korea	Cohort study	Aspirin	6,391	3938/2453	64.3 ± 13.4	NIHSS≤5	e.f	Good
				DAPT (tclopidogrel + aspirin)	5,243	3281/1962	66.7 ± 12.5			
Chen HS et al.	2023	China	RCT	DAPT	369	256/113	64.3 ± 10.4	NIHSS≤5	a.b.c.f	Low
				Alteplase	350	240/110	63.7 ± 11.2			
Gao Y et al.	2023	China	RCT	DAPT	3,050	1987/1063	64.3 ± 10.4	NIHSS≤3	d.e.f	Low
				Aspirin	3,050	1928/1122	64.3 ± 10.4			
Sykora M et al.	2023	Austria	Cohort study	IVT	1,195	751/444	68.1 ± 14	NIHSS≤3	a	Good
				DAPT	2,625	1609/1016	70.8 ± 12			
van der Ende NAM et al.	2023	Netherland	RCT	Mutant Prourokinase + Alteplase	121	69/52	66.9 ± 11.3	NIHSS≤5	a.b.f	Low
				alteplase	117	78/39	68.6 ± 14.3			
Alet MJ et al.	2024	Argentina	Cohort study	SAPT (aspirin or clopidogrel)	27	16/11	63.0 ± 17.0	NIHSS≤5	a.b	Good
				DAPT (aspirin + clopidogrel)	26	20/6	67 ± 16			
Jin X et al.	2024	China	Cohort study	DAPT	363	117/246	66.6 ± 12.7	NIHSS≤5	b.d	Good
				Argatroban	270	93/177	68.4 ± 11.2			
Wang D et al.	2024	China	Cohort study	IVT	492	143/349	64.7 ± 12.0	NIHSS≤5	a.c.f	Good
				DAPT	534	141/393	60.5 ± 12.2			
Jiang Z et al.	2025	China	Cohort study	DAPT	31	18/13	66.0 ± 15.5	NIHSS≤5	a.b	Good
				Tenecteplase	31	8/23	67.5 ± 7.0			

a. mRS 0–1; b. mRS 0–2; c. early neurological improvement at 24 h; d. intracranial hemorrhage; e. newly diagnosed hemorrhagic stroke with 90d; f. all-cause mortality.

### 3.3 Methodological quality of included studies

**Figure 2:**
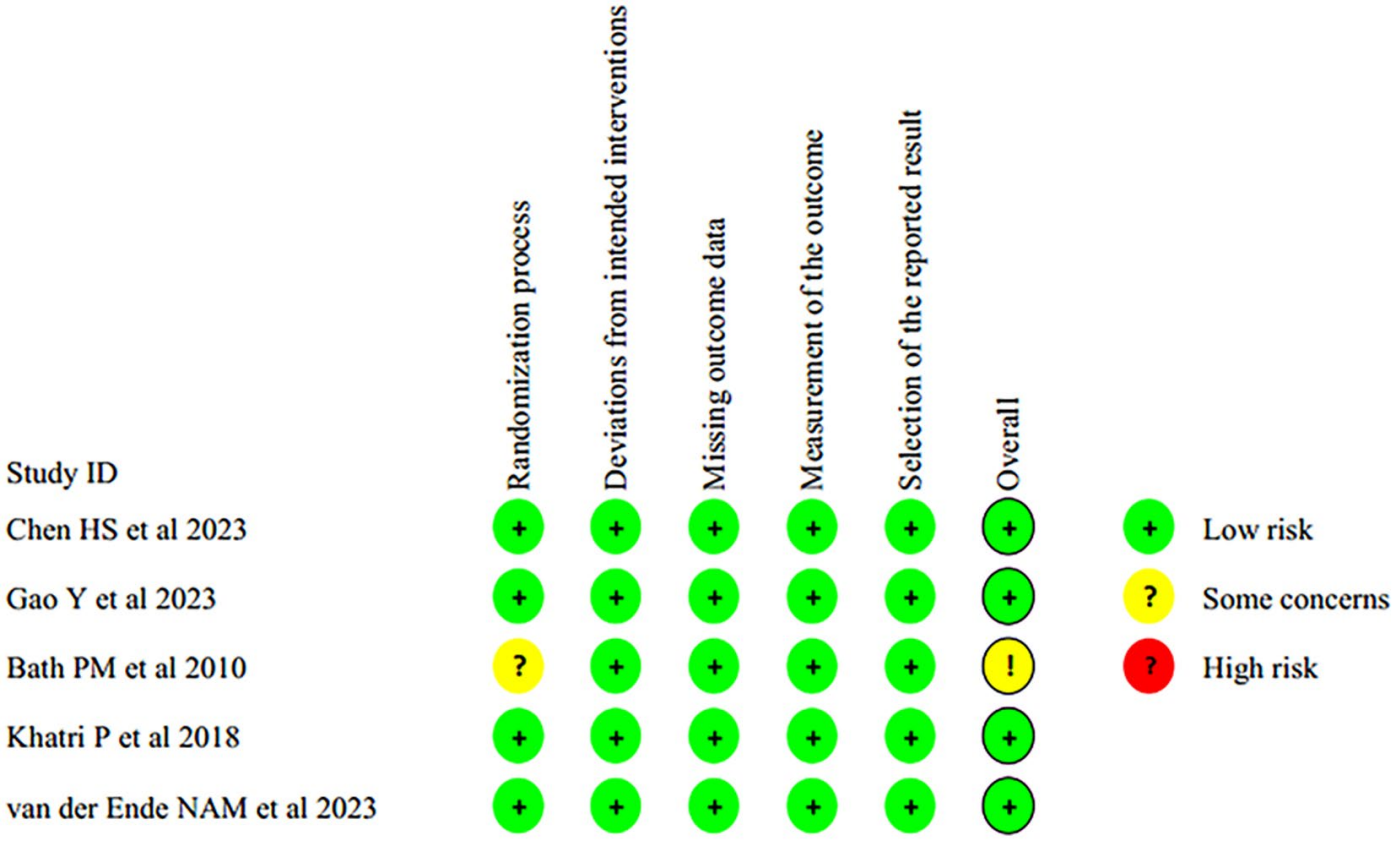
Risk of bias assessment.

The risk of bias assessment for the 5 included RCTs is depicted in Figure 2. Regarding bias from the randomization process, 1 study was rated as having some concerns due to failure to implement random allocation or allocation concealment, while the remaining 4 studies were rated as low risk. All studies were rated as low risk for other domains. Overall, the risk of bias in the included studies was low. The quality of the remaining 7 studies was evaluated using NOS. All these studies scored 8 points and were thus classified as high-quality studies (Supplementary material 3).

### 3.4 Network analysis results

The transitivity assumption was assessed by examining the comparability of patient populations and clinical characteristics across the included studies. All studies enrolled patients with mAIS defined by an NIHSS score of ≤ 3 or ≤ 5 whose age ranges and baseline comorbidities were similar, providing a reasonable clinical basis for the transitivity assumption. Given the limited evidence from RCTs specifically targeting patients with mAIS, the inclusion of both RCTs and cohort studies in the primary network aligns with the methodological guidelines for NMA. All included cohort studies met high-quality criteria and included multivariate adjustments for key confounding factors. Subgroup analyses stratified by study design showed consistent trends across most outcome measures, providing empirical support for the evidence assumption. Nevertheless, residual confounding introduced by the observational study design cannot be completely ruled out, and the results should be interpreted with caution in this context.

#### 3.4.1 Consistency testing

The observed ΔDIC values for all outcome measures in the included studies were below the standard threshold (≤ 5) (Supplementary material 4): mRS (0–1) (ΔDIC = 0.04), mRS (0–2) (ΔDIC = 0.15), early neurological improvement at 24 h (ΔDIC = 0.02), ICH (ΔDIC = 1.04), early neurological improvement at 24 h (ΔDIC = 1.09), and ACM (ΔDIC = 0.89). Consequently, a consistency model was employed for all primary NMAs.

#### 3.4.2 Network diagrams

**Figure 3:**
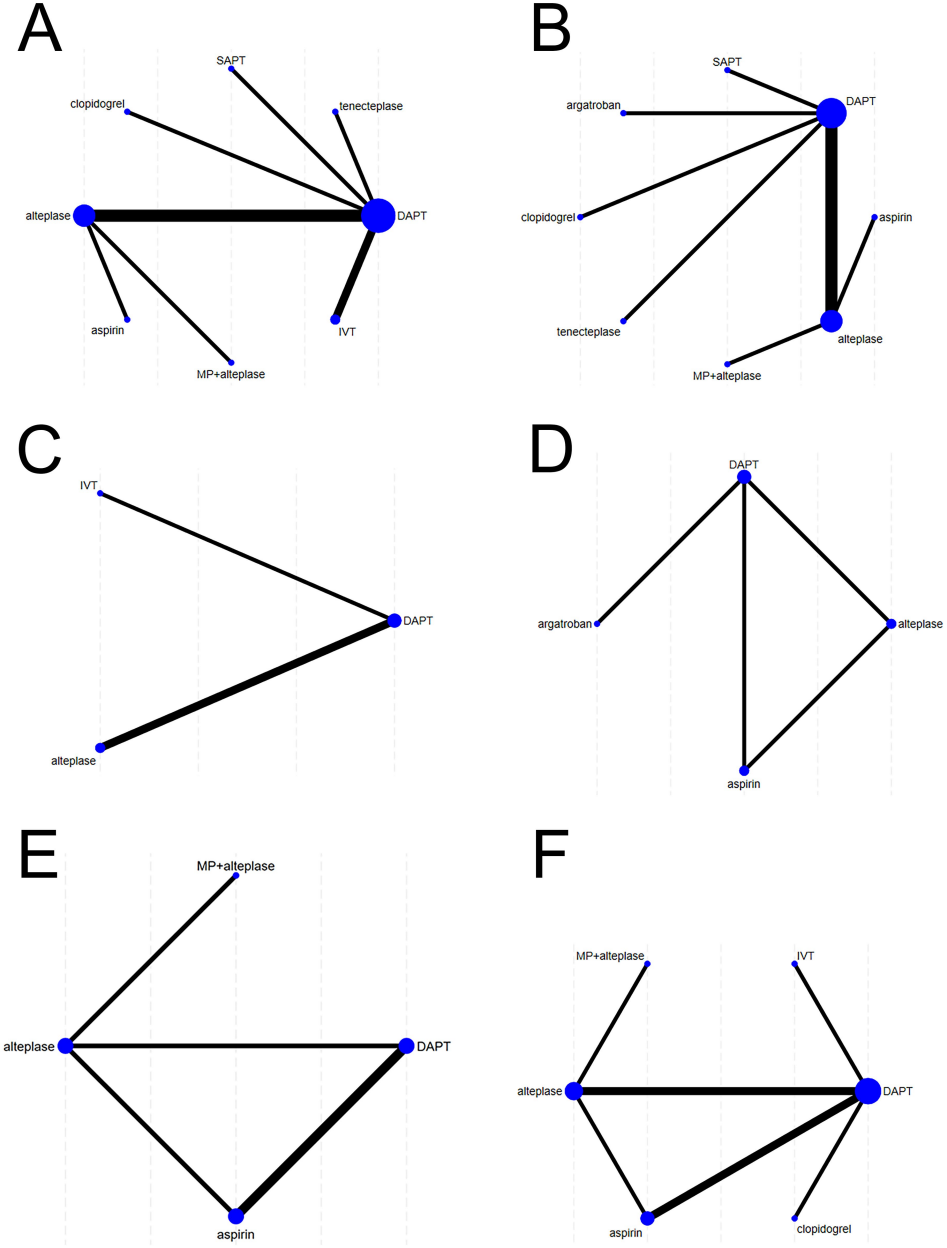
Network analysis diagrams. **(A)** mRS score 0–1; **(B)** mRS score 0–2; **(C)** early neurological improvement at 24 h; **(D)** intracranial hemorrhage; **(E)** newly diagnosed ischemic stroke within 90d; **(F)** all-cause mortality.

The 12 included studies involved 9 interventions, namely aspirin, clopidogrel, SAPT, DAPT (aspirin + clopidogrel), alteplase, IVT, methylprednisolone (MP) + alteplase, and argatroban. IVT encompasses all intravenous thrombolytic agents used in the included studies (alteplase and tinaplase). SAPT refers broadly to monotherapy with antiplatelet agents. Clopidogrel is derived from studies reporting the efficacy of clopidogrel alone, representing a specific SAPT drug. The drug-level nodes listed above are retained as independent interventions to preserve treatment information specific to each drug. Merging them into the corresponding category nodes would result in a loss of information granularity. Therefore, results of indirect comparisons between nodes with a hierarchical relationship (e.g., alteplase versus IVT, clopidogrel versus SAPT) should be interpreted with caution. Figure 3 depicts the network diagrams for the relationships between different interventions. In these diagrams, line thickness is proportional to the number of pairwise comparisons between studies, while node diameter is proportional to the sample size for each intervention. No closed loops were formed in the network of any outcome, which means that all comparisons between interventions relied entirely on indirect evidence via common comparators. There were no direct–indirect evidence pairs available for formal consistency testing. Therefore, node-splitting analysis was not performed, as this method requires the presence of closed loops to distinguish between direct and indirect evidence.

#### 3.4.3 Network analysis results

##### 3.4.3.1 mRS (0–1)

**Figure 4:**
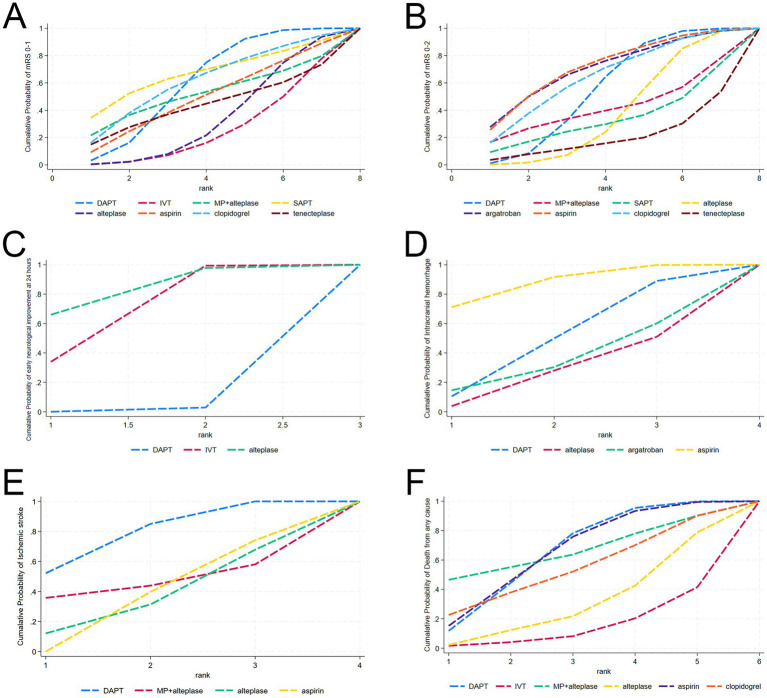
Cumulative probabilities rankograms. **(A)** mRS score 0–1; **(B)** mRS score 0–2; **(C)** early neurological improvement at 24 h; **(D)** intracranial hemorrhage; **(E)** newly diagnosed ischemic stroke within 90d; **(F)** all-cause mortality.

A total of 9 trials involving 7,809 patients reported mRS (0–1). The league table for the NMA showed no significant differences in the outcomes between any pairwise interventions (Table 2). Cumulative probabilities indicated that SAPT (SUCRA = 65.8%), clopidogrel (SUCRA = 60.8%), and DAPT (aspirin and clopidogrel) (SUCRA = 60.7%) ranked as the three most likely best interventions for achieving mRS (0–1) (Figure 4A).

**Table 2:** League table for pairwise comparisons of interventions in achieving mRS (0–1).

**Alteplase**	1.04 (0.78, 1.38)	1.1 (0.78, 1.56)	1.08 (0.89, 1.32)	0.98 (0.74, 1.29)	1.05 (0.71, 1.59)	1.15 (0.74, 1.8)	1.04 (0.66, 1.64)
0.96 (0.72, 1.27)	**Aspirin**	1.05 (0.67, 1.66)	1.03 (0.74, 1.48)	0.94 (0.63, 1.39)	1.01 (0.62, 1.66)	1.1 (0.66, 1.87)	0.99 (0.59, 1.69)
0.91 (0.64, 1.28)	0.95 (0.6, 1.48)	**Clopidogrel**	0.98 (0.74, 1.31)	0.89 (0.63, 1.25)	0.96 (0.57, 1.63)	1.04 (0.64, 1.69)	0.94 (0.58, 1.55)
0.92 (0.76, 1.12)	0.97 (0.68, 1.35)	1.02 (0.76, 1.34)	**DAPT**	0.91 (0.74, 1.09)	0.97 (0.62, 1.54)	1.06 (0.72, 1.58)	0.96 (0.64, 1.45)
1.02 (0.77, 1.35)	1.06 (0.72, 1.58)	1.12 (0.8, 1.58)	1.1 (0.92, 1.35)	**IVT**	1.07 (0.66, 1.76)	1.16 (0.76, 1.83)	1.06 (0.68, 1.67)
0.95 (0.63, 1.42)	0.99 (0.6, 1.62)	1.05 (0.61, 1.75)	1.03 (0.65, 1.61)	0.93 (0.57, 1.51)	**MP+alteplase**	1.09 (0.6, 1.98)	0.99 (0.54, 1.8)
0.87 (0.56, 1.35)	0.91 (0.53, 1.53)	0.96 (0.59, 1.57)	0.95 (0.63, 1.39)	0.86 (0.55, 1.32)	0.92 (0.51, 1.66)	**SAPT**	0.91 (0.51, 1.6)
0.96 (0.61, 1.51)	1.01 (0.59, 1.69)	1.06 (0.65, 1.73)	1.04 (0.69, 1.57)	0.95 (0.6, 1.48)	1.01 (0.56, 1.86)	1.1 (0.63, 1.97)	**Tenecteplase**

This is a league table summarizing pairwise comparisons from the network meta-analysis. The bold text on the diagonal indicates the name of each intervention; these names also serve as the row and column labels for the matrix. Each off-diagonal cell shows the risk ratio (RR) and 95% credible interval (CrI) for the column-defining intervention versus the row-defining intervention.

##### 3.4.3.2 mRS (0–2)

A total of 8 trials involving 3,596 patients reported mRS (0–2). The league table for the NMA showed no significant differences in the outcomes between any pairwise interventions (Table 3). Cumulative probabilities indicated that aspirin (SUCRA = 67.5%), argatroban (SUCRA = 63.8%), and clopidogrel (SUCRA = 63.2%) ranked as the three most likely best interventions for achieving mRS (0–2) (Figure 4B).

**Table 3:** League table for pairwise comparisons of interventions in achieving mRS (0–2).

**Alteplase**	1.04 (0.95, 1.12)	1.04 (0.97, 1.11)	1.03 (0.96, 1.1)	1.01 (0.99, 1.04)	0.99 (0.85, 1.15)	0.98 (0.82, 1.17)	0.95(0.79, 1.11)
0.97(0.89, 1.05)	**Argatroban**	1 (0.9, 1.12)	0.99 (0.9, 1.1)	0.98 (0.91, 1.06)	0.96 (0.8, 1.14)	0.95 (0.78, 1.14)	0.92(0.76, 1.09)
0.96(0.9, 1.03)	1 (0.89, 1.11)	**Aspirin**	0.99 (0.9, 1.09)	0.98 (0.91, 1.05)	0.96 (0.81, 1.13)	0.95 (0.78, 1.14)	0.92(0.76, 1.08)
0.97(0.91, 1.04)	1.01 (0.91, 1.11)	1.01 (0.92, 1.11)	**Clopidogrel**	0.99 (0.93, 1.05)	0.97 (0.82, 1.14)	0.96 (0.79, 1.15)	0.93(0.77, 1.09)
0.99(0.96, 1.01)	1.02 (0.94, 1.1)	1.02 (0.95, 1.1)	1.01 (0.95, 1.07)	**DAPT**	0.98 (0.84, 1.14)	0.97 (0.81, 1.15)	0.94(0.78, 1.09)
1.01(0.87, 1.18)	1.05 (0.88, 1.25)	1.05 (0.89, 1.24)	1.04 (0.88, 1.22)	1.02 (0.88, 1.2)	**MP+alteplase**	0.99 (0.78, 1.24)	0.96(0.76, 1.19)
1.02(0.86, 1.23)	1.06 (0.87, 1.29)	1.06 (0.88, 1.29)	1.05 (0.87, 1.27)	1.03 (0.87, 1.24)	1.01 (0.81, 1.28)	**SAPT**	0.97(0.76, 1.22)
1.05(0.9, 1.26)	1.09 (0.92, 1.32)	1.09 (0.92, 1.32)	1.08 (0.92, 1.3)	1.07 (0.92, 1.28)	1.04 (0.84, 1.32)	1.03 (0.82, 1.32)	**Tenecteplase**

This is a league table summarizing pairwise comparisons from the network meta-analysis. The bold text on the diagonal indicates the name of each intervention; these names also serve as the row and column labels for the matrix. Each off-diagonal cell shows the risk ratio (RR) and 95% credible interval (CrI) for the column-defining intervention versus the row-defining intervention.

##### 3.4.3.3 Early neurological improvement at 24 h

A total of 2 trials involving 1,745 patients reported early neurological improvement at 24 h. The league table for the NMA showed no significant differences in the outcomes between any pairwise interventions (Table 4). Cumulative probabilities indicated that alteplase (SUCRA = 79.8%) had the highest probability of being the most effective in improving neurological function early at 24 h, followed by IVT (SUCRA = 64.7%), while DAPT (SUCRA = 11.5%) exhibited the lowest probability (Figure 4C).

**Table 4:** League table for pairwise comparisons of interventions in achieving early neurological improvement at 24 h.

**Alteplase**	0.79 (0.53, 1.19)	0.94 (0.57, 1.56)
1.26 (0.84, 1.87)	**DAPT**	1.19 (0.87, 1.63)
1.06 (0.64, 1.75)	0.84 (0.62, 1.15)	**IVT**

This is a league table summarizing pairwise comparisons from the network meta-analysis. The bold text on the diagonal indicates the name of each intervention; these names also serve as the row and column labels for the matrix. Each off-diagonal cell shows the risk ratio (RR) and 95% credible interval (CrI) for the column-defining intervention versus the row-defining intervention.

##### 3.4.3.4 ICH

A total of 5 trials involving 7,264 patients reported ICH. The league table for the NMA showed that the rate of ICH was significantly higher with alteplase than with aspirin (RR = 3.05, 95% CrI: 1.21–8.92). Other pairwise comparisons of interventions revealed no significant differences (Table 5). Cumulative probabilities indicated that aspirin (SUCRA = 87.6%) exhibited the greatest likelihood of achieving the lowest rate of ICH, followed by DAPT (SUCRA = 49.8%) and argatroban (SUCRA = 35.0%) (Figure 4D).

**Table 5:** League table for pairwise comparisons of interventions in reducing ICH.

**Alteplase**	0.8 (0.12, 5.03)	0.33 (0.11, 0.82)	0.60 (0.19, 1.77)
1.25 (0.2, 8.03)	**Argatroban**	0.40 (0.08, 2.10)	0.74 (0.17, 3.24)
3.05 (1.21, 8.92)	2.47 (0.48, 13.2)	**Aspirin**	1.83 (0.86, 4.05)
1.67 (0.57, 5.4)	1.35 (0.31, 5.92)	0.55 (0.25, 1.16)	**DAPT**

This is a league table summarizing pairwise comparisons from the network meta-analysis. The bold text on the diagonal indicates the name of each intervention; these names also serve as the row and column labels for the matrix. Each off-diagonal cell shows the risk ratio (RR) and 95% credible interval (CrI) for the column-defining intervention versus the row-defining intervention.

##### 3.4.3.5 Newly diagnosed IS within 90 days

A total of 6 trials involving 18,265 patients reported newly diagnosed IS within 90 days. The league table for the NMA showed that the rate of newly diagnosed IS within 90 days was significantly lower with DAPT than with aspirin (RR = 0.79, 95% CrI: 0.73–0.86) (Table 6). Cumulative probabilities indicated that DAPT (SUCRA = 79.1%) has the highest probability of being the most effective in lowering the rate of newly diagnosed IS within 90 days, followed by MP + alteplase (SUCRA = 45.9%), while alteplase alone exhibited the lowest probability (SUCRA = 37.1%) (Figure 4E).

**Table 6:** League table for pairwise comparisons of interventions in reducing newly diagnosed IS within 90 days.

**DAPT**	1.38 (0.15, 12.46)	1.27 (1.17, 1.38)	1.43 (0.51, 4.00)
0.72 (0.08, 6.53)	**MP+alteplase**	0.92 (0.10, 8.27)	1.03 (0.15, 7.22)
0.79 (0.73, 0.86)	1.09 (0.12, 9.82)	**Aspirin**	1.13 (0.40, 3.15)
0.70 (0.25, 1.96)	0.97 (0.14, 6.75)	0.89 (0.32, 2.48)	**Alteplase**

This is a league table summarizing pairwise comparisons from the network meta-analysis. The bold text on the diagonal indicates the name of each intervention; these names also serve as the row and column labels for the matrix. Each off-diagonal cell shows the risk ratio (RR) and 95% credible interval (CrI) for the column-defining intervention versus the row-defining intervention.

##### 3.4.3.6 ACM

A total of 7 trials involving 21,378 patients reported ACM. The league table for the NMA showed no significant differences between any pairwise interventions (Table 7). Cumulative probabilities indicated that DAPT (SUCRA = 68.2%) exhibited the greatest likelihood of achieving the lowest rate of ACM, followed by MP + alteplase (SUCRA = 64.8%) and aspirin (SUCRA = 59.3%) (Figure 4F).

**Table 7:** League table for pairwise comparisons of interventions in reducing ACM.

**Alteplase**	0.4 (0.03, 3.05)	0.46 (0.03, 6.33)	0.4 (0.04, 2.74)	1.14 (0.07, 15.75)	0.45 (0.04, 3.47)
2.48 (0.33, 29.27)	**Aspirin**	1.17 (0.18, 8.47)	0.96 (0.42, 2.65)	2.83 (0.48, 21.46)	1.1 (0.05, 26.57)
2.16 (0.16, 37.54)	0.85 (0.12, 5.51)	**Clopidogrel**	0.84 (0.15, 4.53)	2.43 (0.23, 28.46)	0.93 (0.03, 32.81)
2.51 (0.36, 26.43)	1.05 (0.38, 2.36)	1.2 (0.22, 6.72)	**DAPT**	2.92 (0.57, 16.79)	1.13 (0.05, 24.65)
0.87 (0.06, 15.36)	0.35 (0.05, 2.09)	0.41 (0.04, 4.39)	0.34 (0.06, 1.74)	**IVT**	0.38 (0.01, 12.68)
2.24 (0.29, 24.74)	0.91 (0.04, 21.26)	1.08 (0.03, 34.55)	0.89 (0.04, 19.56)	2.63 (0.08, 85.16)	**MP+alteplase**

This is a league table summarizing pairwise comparisons from the network meta-analysis. The bold text on the diagonal indicates the name of each intervention; these names also serve as the row and column labels for the matrix. Each off-diagonal cell shows the risk ratio (RR) and 95% credible interval (CrI) for the column-defining intervention versus the row-defining intervention.

##### 3.4.3.7 Convergence diagnostics

Following 50,000 iterations, all NMA models yielded stable and reliable results for all outcome measures. The model estimates shown in Supplementary material 5 remained consistent over time, indicating the credibility of the results.

#### 3.4.4 Heterogeneity analysis

Heterogeneity was assessed using the *I*^2^ statistic. As shown in Supplementary material 6, for mRS (0–1), the pairwise *I*^2^ was 90.99%, while the overall *I*^2^ was 90.99%; for mRS (0–2)*, the pairwise *I*^2^ was 53.04%, while the overall *I*^2^ was 52.97%; and for ACM, the pairwise *I*^2^ was 69.13%, while the overall *I*^2^ was 61.2%. The discrepancies between direct and indirect evidence were consistently below 5%, indicating no significant inconsistency and good model reliability. Furthermore, the heterogeneity was 0% for both early neurological improvement at 24 h and newly diagnosed IS within 90 days, indicating high consistency and good model fit.

### 3.5 Subgroup analyses

#### 3.5.1 Study design

To further investigate the impact of study design on intervention efficacy, subgroup analyses were conducted for RCTs and cohort studies (Supplementary materials 7, 8).

For achieving mRS (0–1), no significant differences were observed in pairwise comparisons between intervention regimens within either the RCT subgroup or the cohort study subgroup. Cumulative probabilities indicated that clopidogrel (SUCRA = 58.6%) was the most likely best regimen in RCTs, while SAPT (SUCRA = 71.1%) was the most likely optimal regimen in cohort studies.

For achieving mRS (0–2), no significant differences were observed in pairwise comparisons between intervention regimens within either the RCT subgroup or the cohort study subgroup. Cumulative probabilities indicated that aspirin (SUCRA = 75.4%) was the most likely best regimen in RCTs, while argatroban (SUCRA = 82.3%) was the most likely optimal regimen in cohort studies.

For reducing ICH, no significant differences were observed in pairwise comparisons between intervention regimens within either the RCT subgroup or the cohort study subgroup. Cumulative probabilities indicated that aspirin (SUCRA = 94.8%) was the most likely best regimen in RCTs, while DAPT (SUCRA = 78.2%) was the most likely optimal regimen in cohort studies.

For reducing newly diagnosed IS within 90 days, DAPT was associated with a significantly lower incidence of newly diagnosed IS within 90 days than aspirin in both the RCT subgroup (RR = 0.76, 95% CrI: 0.64–0.90) and the cohort study subgroup (RR = 0.80, 95% CrI: 0.73–0.88). Cumulative probabilities indicated that DAPT was the most likely optimal regimen for preventing newly diagnosed IS within 90 days in both RCTs (SUCRA = 69.1%) and cohort studies (SUCRA = 94.3%).

For reducing ACM, no significant differences were observed in pairwise comparisons between intervention regimens in either the RCT subgroup or the cohort study subgroup. Cumulative probabilities indicated that aspirin (SUCRA = 73.2%) was the most likely optimal regimen in RCTs, while DAPT (SUCRA = 96.1%) was the most likely optimal regimen in cohort studies.

#### 3.5.2 NIHSS threshold

Although the overall inclusion criteria for this NMA included studies enrolling patients with mAIS (NIHSS ≤ 5), the NIHSS thresholds used for patient enrollment varied across the included studies. Some studies specifically enrolled patients with NIHSS ≤ 3, while others expanded the range and enrolled patients with NIHSS ≤ 5. These differences in NIHSS thresholds across studies may introduce clinical heterogeneity. Therefore, a subgroup analysis by NIHSS thresholds was conducted to investigate whether the NIHSS threshold moderated treatment outcomes. Since only one study was available for the NIHSS ≤ 3 subgroup across most outcome measures, the analysis results are primarily interpretable at the NIHSS ≤ 5 threshold (Supplementary materials 9, 10).

For achieving mRS (0–1), no significant differences were observed in pairwise comparisons between intervention regimens within the NIHSS ≤ 5 subgroup. Cumulative probabilities indicated that SAPT (SUCRA = 66.7%) was the most likely optimal regimen.

For reducing ICH, no significant differences were observed in pairwise comparisons between intervention regimens within the NIHSS ≤ 5 subgroup. Cumulative probabilities indicated that DAPT (SUCRA = 74.2%) was the most likely optimal regimen.

For reducing newly diagnosed IS within 90 days, DAPT was associated with a significantly lower incidence of newly diagnosed IS within 90 days than aspirin in the NIHSS ≤ 5 subgroup (RR = 0.80, 95% CrI: 0.73–0.88). Cumulative probabilities indicated that DAPT (SUCRA = 78.8%) was the most likely optimal regimen.

For reducing ACM, no significant differences were observed in pairwise comparisons between intervention regimens within the NIHSS ≤ 5 subgroup. Cumulative probabilities indicated that DAPT (SUCRA = 76.0%) was the most likely optimal regimen.

#### 3.5.3 Quality rating

To further explore the impact of quality rating on intervention efficacy, subgroup analyses were conducted for studies classified as having low, moderate, or high quality (Supplementary materials 11, 12).

For achieving mRS (0–1), no significant differences were observed in pairwise comparisons between intervention regimens in studies of all quality ratings. Cumulative probabilities indicated that aspirin (SUCRA = 61.8%), clopidogrel (SUCRA = 67.3%), and SAPT (SUCRA = 70.9%) represented the most likely optimal regimen in low-quality studies, moderate-quality studies, and high-quality studies, respectively.

### 3.6 Publication bias

**Figure 5:**
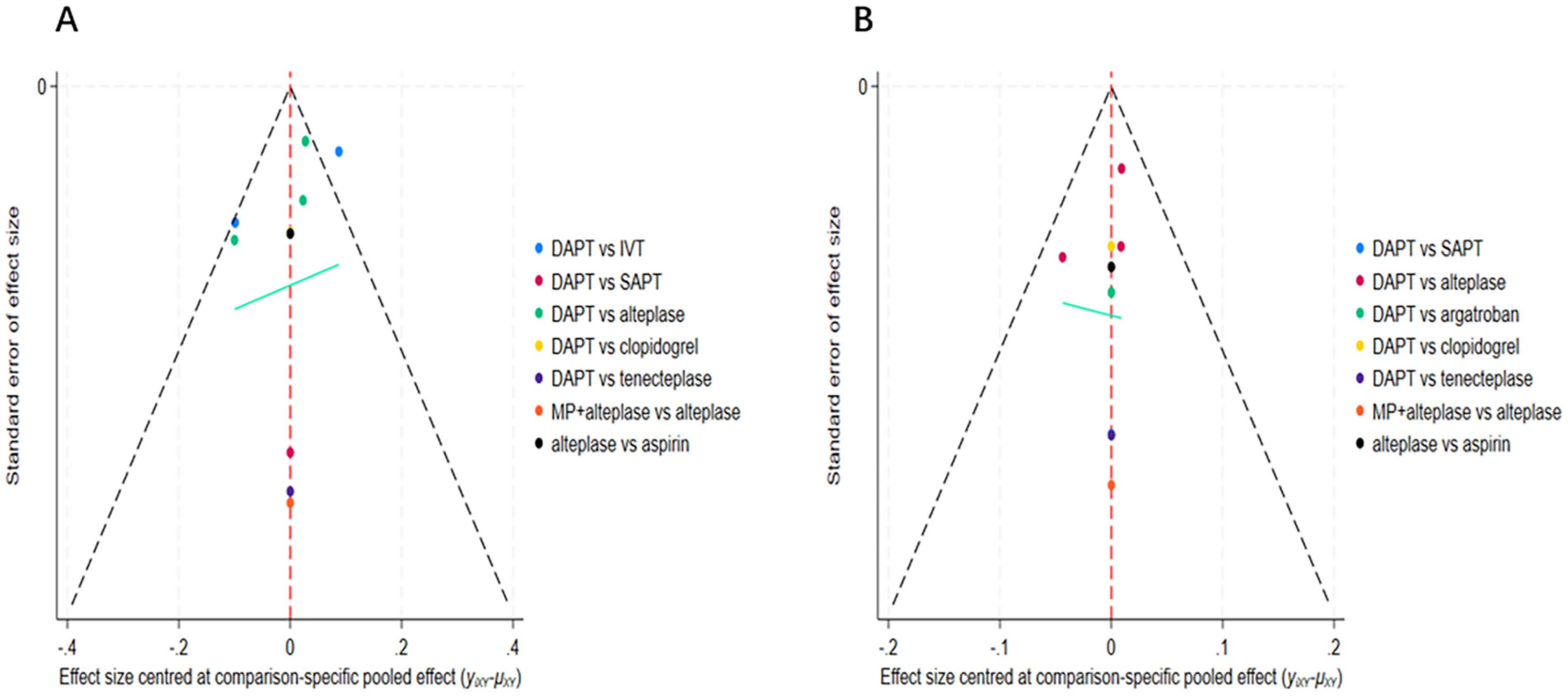
Funnel Plots. **(A)** mRS score 0–1; **(B)** mRS score 0–2.

Funnel plots and Egger’s test were analyzed to evaluate the publication bias for primary outcomes. Egger’s test analysis indicated no evidence of publication bias for mRS (0–1) (*p* = 0.635) and mRS (0–2) (*p* = 0.720) (Figures 5A,B).

## 4 Discussion

### 4.1 Key findings

This NMA systematically synthesized data from 13 eligible RCTs and cohort studies to comprehensively compare the efficacy and safety of various pharmacological interventions for mAIS, including IVT and antiplatelet therapy, in terms of functional outcomes and early neurological changes.

Different pharmaceutical regimens exhibited significant differences in the efficacy for improving 90-day functional outcomes. SAPT (e.g., aspirin or clopidogrel) appeared to perform the best in achieving 90-day functional independence (mRS 0–1). Oral aspirin enteric-coated tablets alone exhibited potentially the best efficacy in achieving 90-day functional independence defined by a broader criterion (mRS 0–2). However, neither of the above differences was statistically significant. They do not constitute evidence of non-inferiority in the formal statistical sense, as this NMA did not establish a predefined non-inferiority margin. Therefore, the above findings should be regarded as hypothesis-generating evidence rather than confirmatory conclusions.

Regarding early neurological function assessment, the optimal interventions varied by time point. Tirofiban was associated with the lowest incidence of early neurological deterioration within 7 days. DAPT was associated with the lowest incidence of early functional deterioration within 24 h. Alteplase exhibited the greatest likelihood of achieving early functional improvement within 24 h and improving 90-day functional outcomes in patients with mAIS. This may be because alteplase, as a recombinant tissue plasminogen activator, is a thrombolytic agent. It activates plasminogen into plasmin. Plasmin promotes the dissolution of thrombi (38), thereby restoring blood flow (39). Consistent with previous studies, these findings suggest that for early fluctuations in the condition of patients with minor stroke, differentiated intervention strategies should be selected based on clinical objectives (e.g., to prevent deterioration or to promote improvement) (40).

Regarding safety and long-term risk management, DAPT was associated with the lowest incidence of newly diagnosed IS within 90 days and a low rate of ICH. Aspirin enteric-coated tablets alone were inferior to DAPT (aspirin + clopidogrel) in reducing newly diagnosed IS within 90 days, but were associated with the lowest rate of ICH. This indicates that despite favorable benefits to patients, risks also exist. However, the SUCRA value reflects the probability that a given treatment regimen ranks at a specific position within the network. It does not in itself constitute evidence of statistical superiority. Therefore, the treatment rankings presented in this study are intended to serve as a reference for the hypothesis of rankings in future research and to provide Supplementary information for clinical decision-making, rather than as definitive recommendations for preferred treatment regimens.

### 4.2 Comparison with previous studies

The findings of this study regarding the efficacy of DAPT regimens are highly consistent with those of classic RCTs such as the CHANCE and POINT studies (41), further validating the reliability of our findings. The CHANCE study enrolled 5,170 patients with mAIS or TIA. Their results have shown that DAPT (aspirin + clopidogrel) for 21 days, followed by sequential antiplatelet monotherapy, significantly reduces the recurrence rate of stroke within 90 days without substantially increasing the risk of major bleeding. The POINT study has also confirmed that DAPT outperforms monotherapy in reducing the recurrence risk of stroke. In our study, the 95% CrI of RR for DAPT regarding the efficacy endpoint of mRS 0–1 was wide due to heterogeneity in the samples. However, the core trend aligns with the above studies, which further supports the efficacy of DAPT in the secondary prevention of minor stroke.

Meanwhile, the effectiveness and safety profile of alteplase in our study also align with a previous guideline (42). According to this guideline, a 2014 meta-analysis (43) of 9 RCTs has shown no significant difference in the benefits of alteplase IVT among patients with minor, moderate, or major stroke. Moreover, among patients with minor disabling stroke, the thrombolytic therapy group demonstrates a higher proportion of favorable outcomes at 3 months. Our study supplemented data from the past 5 years, confirming the superior efficacy of alteplase in early functional improvement within 24 h. Besides, it also confirmed the limitation of alteplase due to a high risk of bleeding, thereby providing updated evidence to support guideline recommendations.

A large NMA (44) published in 2023 (encompassing 37 studies and 302,114 patients with newly diagnosed IS) has reported a 4.2% recurrence rate of stroke within 90 days and a peak of recurrence within 7 days. Likewise, our study exhibited a consistent overall trend regarding the recurrence rate of stroke within 90 days. However, differences existed in the ranking of specific interventions by efficacy. For instance, the 2023 NMA has demonstrated superior effectiveness of aspirin + ticagrelor over aspirin + clopidogrel in preventing the recurrence of stroke within 90 days. However, due to the limited number of RCTs involving ticagrelor included in our study (only two small-sample studies), this conclusion could not be fully validated. Another study, Qiu et al. (45) investigating AIS of varying severity has suggested that aspirin + ticagrelor has the highest probability of being the optimal regimen for minor stroke. In our study, however, DAPT (aspirin + clopidogrel) exhibited the greatest likelihood of being the best intervention for preventing the recurrence of stroke. The core reason for this discrepancy is the differences in study populations and inclusion criteria between the previous NMA and our study. The former NMA has not strictly defined the threshold of the NIHSS score for minor stroke (some studies enrolled patients with NIHSS scores ≤ 8) and has included more patients with cardioembolic stroke. Our study strictly limited NIHSS scores to ≤ 5 and primarily included patients with non-cardiogenic stroke. This discrepancy suggests that stratification of stroke severity and etiological classification may represent key confounding factors influencing the efficacy of interventions. Hence, more refined subgroup analyses are warranted in future studies.

Additionally, our study revealed a lower rate of ICH with DAPT than with alteplase. This finding does not align with the conclusions of some previous NMAs. Most prior studies have not identified significant differences between these two interventions. This discrepancy may be attributed to the high proportion of patients at high risk for bleeding in the studies investigating alteplase included in our analysis (e.g., 2 studies enrolled patients aged > 80 years or with a history of hypertension). Therefore, the impact of baseline stratification of bleeding risk on outcomes is a critical consideration in clinical practice.

The high heterogeneity observed in the mRS (0–1) outcome may stem from the inclusion of both RCTs and cohort studies, differences in the NIHSS thresholds used to define mild stroke across studies (≤ 3 versus ≤ 5), differences in the timing of treatment initiation, and variations in stroke etiology and concomitant medication use across studies. Nevertheless, the ΔDIC values for this outcome remained well below the generally accepted threshold, and the results of the subgroup analysis stratified by study design showed consistent effect directions, collectively supporting the robustness of the main conclusions.

### 4.3 Clinical value

Patients with mAIS (NIHSS ≤ 5) present with relatively mild symptoms but a high risk of early recurrence. The optimal pharmacological treatment strategy remains controversial. This NMA systematically compared the relative effectiveness and safety of various antithrombotic therapies (e.g., SAPT, DAPT, anticoagulation) by combining direct and indirect evidence, thereby addressing the limitations of head-to-head randomized trials. This study provides clinicians with a comparative overview of current pharmacological treatment regimens, suggesting that DAPT may offer potential benefits in the short-term prevention of stroke recurrence. However, this observation is primarily based on statistically significant pairwise comparisons (e.g., DAPT versus aspirin in terms of newly diagnosed IS) and should be interpreted in conjunction with individual patient characteristics and clinical judgment. This ranking guides individualized decision-making and secondary prevention strategies for stroke, thereby reducing recurrence risk while avoiding the harm of bleeding associated with overtreatment. In summary, this study is potentially valuable for enhancing the quality of minor stroke management.

### 4.4 Limitations

Despite strict adherence to the methodological norms for systematic review and NMA, limitations exist in this study. First, the primary network incorporates both RCTs and cohort studies, which differ substantially in their susceptibility to bias and confounding. Although this approach was justified by the scarcity of RCT-level evidence specifically targeting the mAIS population, and supported by the directional consistency observed in the subgroup analysis stratified by study design, the inclusion of observational evidence introduces residual confounding that cannot be fully accounted for. In particular, treatment selection bias inherent to cohort studies may distort effect estimates in the primary network. The subgroup analysis restricted to RCTs, therefore, represents a more conservative and internally valid evidence stratum, and should be prioritized when interpreting findings with direct clinical implications. In addition, mixed study designs, variability in NIHSS thresholds, and differences in treatment time windows contribute to residual heterogeneity across outcomes that cannot be fully eliminated through subgroup analyses. Second, the number of included studies and participants varies considerably across interventions, with studies investigating DAPT providing substantially stronger evidence than those evaluating alteplase, argatroban, or MP + alteplase. This imbalance may introduce differential precision in effect estimates and disproportionately influence SUCRA rankings. Findings for interventions supported by limited evidence should be regarded as preliminary, pending confirmation from dedicated trials. Third, although interventions are ranked by SUCRA, overlapping CrIs are observed across multiple outcomes. For outcomes where no statistically significant pairwise differences were identified, including mRS (0–1), mRS (0–2), and ACM, SUCRA rankings should be regarded as hypothesis-generating only, rather than confirmatory evidence of treatment superiority. Fourth, the publication bias assessment exhibits significant limitations in this network. Egger’s test has limited statistical power in small networks, and in the absence of closed loops, network-level bias tests cannot be applied. The absence of detected bias should not be interpreted as confirmation that publication bias is absent. Fifth, SUCRA-based rankings derived from weakly connected networks, where most comparisons rely on indirect evidence, are subject to considerable uncertainty and should be regarded as hypothesis-generating rather than definitive clinical guidance. Sixth, given individual patient variability and the complexity of clinical practice, the conclusions of this NMA represent average effects and cannot fully reflect differences among individual patients. Patients with mAIS exhibit significant variability in age, sex, comorbidities, stroke etiology, and bleeding risk scores (e.g., HAS-BLED, PRECISE-DAPT), all of which influence treatment response. The optimal regimen identified in this study, therefore, serves only as a reference for clinical decision-making and cannot replace individualized treatment choices.

## 5 Conclusion

This study suggested that SAPT, aspirin, and alteplase had the highest probability of being the most effective for improving outcomes of mRS (0–1), mRS (0–2), and early neurological function at 24 h. Among these, aspirin was also associated with the lowest ICH rate, while DAPT was associated with the lowest rates of newly diagnosed IS and ACM within 90 days. However, for most other outcome measures, no statistically significant differences were found among the interventions. Therefore, findings based on rankings should be interpreted with caution. These findings indicate that individualized treatment strategies should be developed based on patient characteristics. Furthermore, given the limitations in sample size, future high-quality, large-scale RCTs are warranted to further examine these findings.

## Data Availability

The original contributions presented in the study are included in the article/Supplementary material, further inquiries can be directed to the corresponding author.
